# Arginine and antioxidant supplement on performance in elderly male cyclists: a randomized controlled trial

**DOI:** 10.1186/1550-2783-7-13

**Published:** 2010-03-23

**Authors:** Steve Chen, Woosong Kim, Susanne M Henning, Catherine L Carpenter, Zhaoping Li

**Affiliations:** 1 Center for Human Nutrition, David Geffen School of Medicine at UCLA, Los Angeles, CA 90095, USA

## Abstract

**Background:**

Human exercise capacity declines with advancing age. These changes often result in loss of physical fitness and more rapid senescence. Nitric oxide (NO) has been implicated in improvement of exercise capacity through vascular smooth muscle relaxation in both coronary and skeletal muscle arteries, as well as via independent mechanisms. Antioxidants may prevent nitric oxide inactivation by oxygen free radicals. The purpose of this study was to investigate the effects of an L-arginine and antioxidant supplement on exercise performance in elderly male cyclists.

**Methods:**

This was a two-arm prospectively randomized double-blinded and placebo-controlled trial. Sixteen male cyclists were randomized to receive either a proprietary supplement (Niteworks^®^, Herbalife International Inc., Century City, CA) or a placebo powder. Exercise parameters were assessed by maximal incremental exercise testing performed on a stationary cycle ergometer using breath-by-breath analysis at baseline, week one and week three.

**Results:**

There was no difference between baseline exercise parameters. In the supplemented group, anaerobic threshold increased by 16.7% (2.38 ± 0.18 L/min, p < 0.01) at week 1, and the effect was sustained by week 3 with a 14.2% (2.33 ± 0.44 L/min, p < 0.01). In the control group, there was no change in anaerobic threshold at weeks 1 and 3 compared to baseline (1.88 ± 0.20 L/min at week 1, and 1.86 ± 0.21 L/min at week 3). The anaerobic threshold for the supplement groups was significantly higher than that of placebo group at week 1 and week 3. There were no significant changes noted in VO_2 _max between control and intervention groups at either week 1 or week 3 by comparison to baseline.

**Conclusion:**

An arginine and antioxidant-containing supplement increased the anaerobic threshold at both week one and week three in elderly cyclists. No effect on VO_2 _max was observed. This study indicated a potential role of L-arginine and antioxidant supplementation in improving exercise performance in elderly.

## Introduction

Human exercise capacity declines with advancing age and many individuals lose the inclination to participate in regular physical activity. These changes often result in loss of physical fitness and more rapid senescence. A dietary supplement that increases exercise capacity might preserve physical fitness and improve general health and well being in older humans.

Endothelial nitric oxide synthase (eNOS) uses the amino acid L-arginine as a substrate to synthesize nitric oxide (NO). When released from endothelium cells, NO can dilate arteries to increase blood flow [1], help maintain endothelial elasticity [2], prevent platelets from adhering to artery walls [3], mediate erections through smooth muscle relaxation [4], and increase capacity for exercise [5]. In addition, NO can play an integral part in the immune system [6], assist in memory function [7] and sleep regulation [8]. It should also be noted that in general, youthful, healthy and athletic individuals have a healthier eNOS system, compared to sedentary, unhealthy and aging individuals [9]. A healthy NO and vascular system facilitates the healthy function of arterioles that mediate oxygen delivery to multiple organs and tissues, including the muscles and kidneys that may impact exercise performance [10].

NO production diminishes in quantity and availability as we age and is associated with an increased prevalence of other cardiovascular risk factors [11]. Hypertension has been shown to promote premature aging of the endothelial system in humans [11]. In individuals with cardiovascular risk factors including hypertension, hypercholesterolemia, smoking, diabetes, obesity, insulin resistance, erectile dysfunction, and metabolic changes associated with aging, supplementation with arginine has been shown to improve NO-dependent endothelial relaxation [12], and improving age-associated endothelial dysfunction [13].

Antioxidants may prevent nitric oxide inactivation by oxygen free radicals. For example, Vitamin C has been shown to improve impaired endothelial vasodilation in essential hypertensive patients, and effect that can be reversed by the nitric oxide synthase inhibitor N^G^-monomethyl-L-arginine[14]. There is also research indicating that the combination of vitamin C, vitamin E (1.0% to water) and L-arginine works synergistically to enhance nitric oxide production, through nitric oxide synthase gene expression[15]. A study in *Atherosclerosis *showed Vitamin E (1000 IU/day) improved endothelium health and increased eNOS expression in hypercholesterolemic subjects [16].

Therefore, the present study was designed to extend the above observations by testing the hypothesis that arginine and antioxidants in combination would enhance performance as indicated by objective measures in a prospectively randomized, placebo-controlled trial in elderly cyclists.

## Methods

### Human subjects

The experimental protocol was approved by the Institutional Review Board at the University of California, Los Angeles. All subjects were informed of the potential risks, benefits, and time requirements prior to signing a written informed consent.

Sixteen male cyclists were recruited to participate in the study through a cycling club in the West Los Angeles area. Men between the ages of 50 and 73 who performed at least 4 hours per week of moderate to intense cycling were screened for this study. Key exclusion criteria included smoking, a history of coronary heart disease, morbid obesity (BMI > 40), or any prior or current medical problems that would limit the subject's physical performance. The participants were apparently healthy and free of any significant medical problems. They were also not taking any medications that impact eNOS system, or other sports enhancing supplementations during the time of the study.

### Study design

This was a three-week, randomized, double-blinded, placebo-controlled clinical intervention trial. During the screening visit, a history and a physical examination were performed. Baseline blood tests including a complete blood count, a routine chemistry panel, and a measurement of cholesterol were also obtained. All subjects underwent baseline exercise testing. If the subjects showed any evidence of ischemic heart disease based on EKG criteria, pulmonary or musculoskeletal diseases that prevented them from finishing the test, they were excluded from the study. If subjects qualified for the study, they were randomized to either the placebo or the supplementation group in a 1:1 ratio. The supplementation began at week 0 after the baseline exercise testing. The subjects returned to the study center at week 1 and week 3 for further exercise testing.

### Performance Assessment

At the initial screening visit, aerobic capacity and physical fitness were assessed by measuring maximal oxygen uptake (VO_2max_) and the gas exchange anaerobic threshold (VO_2θ_) during a symptom limited, incremental work rate exercise test, targeted to last between 8 to 12 minutes. Screening allowed for determination of whether the subject was physically fit to complete the study, could tolerate the experimental setup (including breathing through the mouthpiece), and permitted the subject to accustom to the study protocol. On subsequent visits, exercise endurance was assessed by measuring time to exhaustion at 60% of the maximal work rate achieved during the initial incremental work rate exercise test, with a targeted duration of testing between 45 minutes and 1 hour.

### Incremental Work Rate Exercise Test (IWR) for VO_2max_

Maximal exercise performance was assessed using a symptom-limited incremental exercise protocol on a cycle ergometer [Ergoline 900S; Sensormedics Corp, Loma Linda, CA]. The external work rate was continuously incremented in "ramp" fashion by computer control. The rate of incrementation was judged for each individual subject by considering age, gender, height, weight, and level of habitual exercise activity with the intention of obtaining an exercise phase of 8-12 minutes before exhaustion [17]. The increment in resistance for baseline test and two subsequent tests for each subject was consistent.

Minute ventilation was measured using a mass flow meter; expired fractional concentrations of oxygen and carbon dioxide were continuously monitored by a paramagnetic oxygen analyzer and a non-dispersive infra-red CO2 analyzer, respectively [2900; SensorMedics Corp, Loma Linda, CA].

A 12-lead electrocardiogram was obtained at rest and every two minutes throughout exercise [Quinton 5000; Seattle, WA]; heart rate was monitored continuously by rhythm strip.

### Constant Work Rate Exercise Tests (CWR)

At baseline and final visits, subjects performed a constant work rate (CWR) exercise test at 60% of their maximal work rate determined from the initial IWR test. The experimental setup and monitoring for the CWR tests was identical to the IWR tests.

Subjects arrived at the same time of the day for the baseline and subsequent two visits. They were given general instructions regarding what to eat and/or drink for breakfast on the day of each study, and reminded to ingest the same breakfast each time, so as to minimize variability due to glycemic status and/or time of day. During the endurance exercise test, cumulative oxygen uptake and carbon dioxide output were tracked. Following the test, lactate recovery was measured by earlobe prick lactate analysis at exhaustion and every 3 minutes afterwards up to 12 minutes [Accutrend Lactate, Sports Resource Group, Hawthorne, New York].

When the subject signaled his desire to end the exercise (time of exhaustion), a button on the computer immediately converted the work rate to unloaded pedaling (no resistance) for a recovery period. Endurance was defined as the duration of the CWR exercise to the point of fatigue and expressed as total work performed.

Detection of the anaerobic threshold for lactate accumulation by non-invasive gas exchange measurements is inevitably subject to the possibility of observer error. In order to overcome this difficulty, we separately coded each of the sets of gas exchange data and presented them to two experienced exercise physiologists who were blinded to the study design. A standardized approach to interpretation was agreed beforehand by these observers and has been previously validated [18].

### Supplementation Protocol

The proprietary supplement Niteworks^® ^was manufactured by Herbalife International Inc. (Century City, California, USA). Each serving contained 5.2 g L-arginine in a proprietary blend with L-citrulline, 500 mg ascorbic acid, 400IU vitamin E, 400 μg folic acid, 300 mg L-taurine, and 10 mg alpha lipoic acid in a lemon-flavored powder form. One serving of supplement powder was mixed with 8 oz of water, administered at bedtime based on the rationale that nitric oxide levels are lowest during sleep due to inactivity, lack of food and low blood pressure [19,20]. The placebo group received a powder with all active ingredients replaced with M-100 maltodextrin.

### Blood Tests

Complete blood count, routine chemistry panel, and fasting cholesterol were drawn from the subjects as part of the screening visit. Reduced and oxidized gluthathione levels were measured at each visit before and after the exercise testing in whole blood using the Bioxytech GSH/GSSG-412 kit from Oxis Research (Portland, OR).

### Statistical and Data Analysis

The data was analyzed by one single observer who was blinded and has had experience obtaining the threshold. The results were verified by the investigator.

All measurements were summarized using mean, standard deviation, median, minimum and maximum for each group at each time point. To summarize changes using mean and standard deviation for each group and at each time point, paired t-tests were used to evaluate whether change is different from baseline within each treatment group. Mixed model repeated measures analysis of variance was used to evaluate changes between groups, and the interaction between changes from baseline according to group. SAS statistical software, version 9.1 was used to perform all analyses. All tests were two-sided with significance level 0.05.

## Results

Sixteen cyclists were randomized to two arms (n = 8 in each arm) and all completed the study without any side effects. There were no significant differences in subject demographics. The supplementation group had 8 Caucasian and the placebo group consisted of 7 Caucasian and one African American. The supplementation group's age ranged from 50 to 62 years with an average age of 57.6 years. The placebo group's age ranged from 50 to73 years with an average age of 60.6 years. The weight, height, BMI, blood pressure, resting heart rate, blood count, and metabolic parameters including cholesterol were not statistically different between the two groups of subjects. There were no significant differences in baseline exercise parameters between the two groups (Table 1) including anaerobic threshold (2.04 ± 0.26 L/min and 1.89 ± 0.16 L/min in the placebo and supplemented groups, respectively).

**Table 1:** Subject baseline characteristics

	Supplementation	Placebo
Male	8	8

Race		

African American	0	1

Caucasian	8	7

Age (years) mean ± SD	57.6 ± 4.6	60.6 ± 8.7

Height (inches)	70.6 ± 2.1	70.1 ± 1.4

Weight (pounds)	171.0 ± 16.4	170.6 ± 18.3

BMI (kg/m^2^)	24.1 ± 2.2	24.4 ± 2.9

SBP (mmHg)	111.9 ± 9.2	117.5 ± 9.6

DBP (mmHg)	75.0 ± 7.6	75.6 ± 7.8

Pulse (beats/min)	56.0 ± 6.5	56.0 ± 11.1

Glucose (mg/dL)	77.1 ± 11.7	81.1 ± 19.1

Hgb (g/dL)	14.6 ± 0.8	14.4 ± 0.8

**Figure 1:**
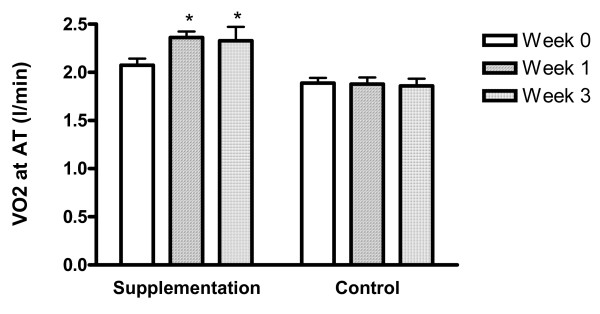
Anaerobic Threshold *p-value < 0.05 between supplementation and control group.

**Figure 2:**
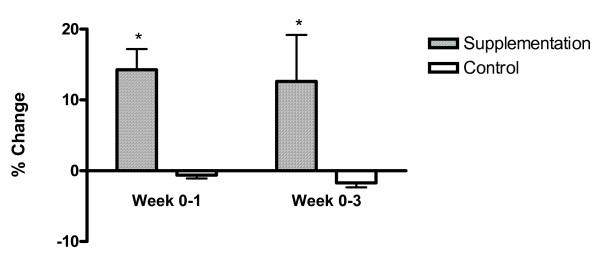
Change in Anaerobic Threshold *p-value < 0.05 between supplementation and control group.

After one week of study, the anaerobic threshold of the supplement group increased to 2.38 ± 0.18 L/min (an increase of 0.34 ± 0.061 L/min with a p-value of < 0.01), while the anaerobic threshold of the control group marginally changed and was not significant This increase in anaerobic threshold was preserved at week 3 with an average increase of 0.29 ± 0.06 L/min in the supplement group (for a total threshold of 2.33 ± 0.40 L/min), while there was no change in the control group (p = 0.21). Therefore, anaerobic threshold in the supplement group increased by 16.7% over baseline at week one and 14.2% over baseline at week three, respectively. (Figure 1, 2 and Table 2).

**Table 2:** Anaerobic Threshold and VO_2max_

	AT (L/min)		VO_2max _(L/min)	
	**Supplementation**	**Placebo**	**Supplementation**	**Placebo**

Week 0	2.04 ± 0.28	1.88 ± 0.16	3.71 ± 0.34	3.22 ± 0.62

Week 2	2.38 ± 0.18*	1.84 ± 0.18	3.69 ± 0.23	3.26 ± 0.46

Week 3	2.33 ± 0.44*	1.83 ± 0.21	3.72 ± 0.27	3.39 ± 0.47

Mean ± SE, *p-value < 0.05 between baseline and week 1, baseline and week 3

We evaluated between group differences for anaerobic threshold values at each time point. At week 1 (p = 0.01) and week 3 (p = 0.02), significant between group differences were observed with supplementation means significantly higher than anaerobic threshold placebo means. We observed a significant interaction between group differences and change from baseline (p = 0.04). Minimal differences for power output (measured in watts) over time compared to baseline and minimal differences between placebo and supplementation were observed (interaction p value = 0.12).

While there was not significant change for the control group, the supplement group had a power output at week 1 of 177.12 ± 21.13 watts as compared with baseline of 154.62 ± 23.21 W. At week three, the increase of power output was sustained at 175.27 ± 36.61 W. This translated to an increase of 22.51 watts at week 1 and 20.66 watts at week 3 (p-value < 0.01).

The VO_2max _results are shown in table 2. There was not any significant change from baseline at neither week 1 nor 3 for either group. Other exercise measurements of blood pressure recovery, pulse recovery, peak lactate, lactate recovery, were not statistically between the supplemented and control groups. There were no changes observed for oxidized glutathione between the two groups or over time.

## Discussion

The role of nitric oxide in cardiovascular health has been well described in literature. The effect of nitric oxide on exercise performance, however, has not been clearly elucidated. During a 5 week progressive strength training program, volunteers were given a supplement containing 1 g arginine and 1 g ornithine, or a placebo, each day. The results suggest that the combination of arginine and ornithine taken in conjunction with a high intensity strength training program can significantly increase muscle strength and lean body mass [21]. Campbell et al [22] observed that arginine and α-ketoglutarate positively influenced 1 RM bench press and Wingate peak power performance in trained adult men. Arginine was also reported to improve peak power significantly in non-athlete men [23]. Conversely, a number of studies have failed to identify any beneficial effect of arginine supplementation. Liu et al [24] investigated the effect of three day supplementation of 6 gram of arginine on performance in intermittent exercise in well-trained male college judo athletes and found the supplementation had no effect on performance. Similarly, it has been shown that supplementation of arginine aspartate for 14 days prior to marathon run did not affect the subsequent performance in trained runners [25].

In the present study, we demonstrated a statistically increase of 16.7% in AT after one week of supplementation with L-arginine and antioxidants. The observed increase in AT was further validated by the increase of 22.51 watts of power output at AT. Based on our data, the supplementation group increased their power output at threshold. Therefore, these physiological changes should be associated with prolonged exercise and a higher work rate due to arginine and antioxidant supplementation. These data obtained were also remarkable in that every subject in the supplemented group demonstrated increases in anaerobic threshold, while none of the subjects in the placebo group demonstrated any increase.

Youthful, healthy, athletic individuals generally have a healthier NO system, compared with aging, unhealthy, sedentary individuals [9]. In humans, exercise capacity declines with advancing age and many individuals lose the inclination to participate in regular physical activity. In healthy adults, arginine can be synthesized in sufficient quantities to meet most normal physiological demands with the rate of de novo synthesis remaining unaffected by several days of an arginine free diet [26,27]. Our study subjects had an average age >55 years, while other studies included young athletes [24,25]. This difference may explain the significant improvement on AT in our study.

As in other studies [26,28] we did not see an increase in VO_2max_, which is defined as the highest value of minute ventilation attained and measured during incremental exercise despite the increase in anaerobic threshold. A possible reason for this lack of increase could be the fact that VO_2max_, as its name implies, is also a maximum effort measurement and, therefore, is effort dependent. By contrast, anaerobic threshold is a more sensitive test to measure changes in exercise performance because it is a submaximal exercise measure that is not effort-dependent. In a recent review in Journal of Applied Physiology [28], Saltin stated that VO_2max _is limited by cardiac output. With the current study design, we would not expect to see an increase in VO_2max _because there is no reason for the cardiac output to increase in these athletes.

It is unclear whether the increase in AT that we observed in this study was due to L-arginine alone, or a combination of the nutrients. Pre-treatment with vitamins C and E has been shown to block vascular dysfunction caused by a high-fat and high-sugar diet [29]. L-arginine, vitamin C, and vitamin E promote a healthy cardiovascular system by supporting enhanced NO production [15]. NO formation is further increased by the recycling effect of L-citrulline to L-arginine and the fact that L-citrulline is taken up into cells by a mechanism independent of that for arginine [30].

This study was performed in trained athletes who were without any cardiovascular problems. The role of L-arginine supplementation in cardiac patients remains controversial. Furthermore, it is also unclear if arginine supplementation in the sedentary population can have the same results. Further research will be needed to assess the interaction of these factors and to determine the effects of prolonged administration of arginine and antioxidants on exercise performance.

## Conclusion

An arginine and antioxidant-containing supplement increased the anaerobic threshold and the work at anaerobic threshold at both week one and week three in elderly cyclists. No effect on VO_2max _was observed. This study indicates a potential role of L-arginine and antioxidant supplementation in improving exercise performance in elderly.

## Competing interests

The authors declare that they have no competing interests.

## Authors' contributions

SC participated in the design of the study and performed the exercise protocol. WK performed the exercise testing protocol. SH analyzed blood samples for glutathione levels. CLC performed statistical analysis. ZL participated in the design of the study protocol, coordination and draft of the manuscript.

All authors have read and approved the final manuscript.
